# gcCov: Linked open data for global coronavirus studies

**DOI:** 10.1002/mlf2.12008

**Published:** 2022-03-16

**Authors:** Wenyu Shi, Guomei Fan, Zhihong Shen, Chuan Hu, Juncai Ma, Yuanchun Zhou, Zhen Meng, Songnian Hu, Yuhai Bi, Liang Wang, Haiying Yu, Siru Lin, Xiuqiang Sun, Xinjiao Zhang, Dongmei Liu, Qinlan Sun, Linhuan Wu

**Affiliations:** ^1^ Microbial Resource and Big Data Center, Institute of Microbiology Chinese Academy of Sciences Beijing China; ^2^ Computer Network Information Center, Chinese Academy of Sciences Beijing China; ^3^ State Key Laboratory of Microbial Resources, Institute of Microbiology Chinese Academy of Sciences Beijing China; ^4^ CAS Key Laboratory of Pathogenic Microbiology & Immunology, Institute of Microbiology Chinese Academy of Sciences Beijing China

## Abstract

We present a method of mapping data from publicly available genomics and publication resources to the Resource Description Framework (RDF) and implement a server to publish linked open data (LOD). As one of the largest and most comprehensive semantic databases about coronaviruses, the resulted gcCov database demonstrates the capability of using data in the LOD framework to promote correlations between genotypes and phenotypes. These correlations will be helpful for future research on fundamental viral mechanisms and drug and vaccine designs. These LOD with 62,168,127 semantic triplets and their visualizations are freely accessible through gcCov at https://nmdc.cn/gccov/.

In recent decades, a family of enveloped positive‐sense RNA viruses, known as coronaviruses (CoVs), have caused severe infectious diseases and posed a continuous global threat to public health^1^. This has resulted in extensive research on novel human and animal CoVs, particularly in the fields of vaccine and therapy development. During 2020, there were over 10,000 publications on COVID‐19 and the number of such publications is still rapidly increasing. This rapid expansion in data has inevitably led to the great challenge of integrating diverse types of studies into a single searchable correlated source. Currently, available CoV databases mainly focused on genomic analysis (e.g., CovDB^1^ and ViPR^2^) or publications (e.g., LitCovid^3^), or mainly focused on SARS‐CoV‐2 (2019nCoVR^4^, ^5^). However, these databases did not establish correlations between genomic data and other types of information (e.g., papers, patents, and antibodies).

As a result, further knowledge discovery is hampered by the inability to investigate interrelationships among these mass data. The semantic web is a promising solution for biomedical data integration. A feature of the semantic web is its ability to integrate distributed web resources into a knowledge base of shared ontologies and then analyze those data to identify underlying relationships between various entities. This enables correlation analyses to be conducted among genomics, structure, antibody, and publication data.

In this study, we integrated data from different publicly available resources and mapped them into a semantic web framework to construct the gcCov database. Then, the gcCov database provides extensive information and relationships regarding CoVs using linked open data (LOD). It helps scientists to detect connections between the linked data and hence to discover new knowledge that would otherwise be hidden in the mass data. To the best of our knowledge, gcCov is the first and only CoV database published using LOD and based on a semantic web framework.

## User interface and visualization of LOD

The gcCov database performs dynamic statistics on updated data resources of nucleotide and protein sequences, three‐dimensional (3D) structures, and literature and patents integrated from various data sources and data formats. We provide a map of CoV distribution based on data from multiple collection sites. Such maps can be used to evaluate CoV infection spread in different countries over the years. Nearly 30 CoVs have been recognized to infect humans, mammals, and other animals. Currently, seven different CoV strains are known to infect humans (HCoVs): HCoV229E (229E), HCoV‐OC43 (OC43), severe acute respiratory syndrome CoV (SARS‐CoV), HCoV‐NL63 (NL63), HCoV‐HKU1 (HKU1), Middle East respiratory syndrome CoV (MERS‐CoV), and the most recent severe acute respiratory syndrome CoV 2 (SARS‐CoV‐2) (also known as COVID‐19)^6^. As a result, data were further organized by HCoVs types. Furthermore, the gcCov portal provides direct results from online data search, as well as data visualization based on semantic relationships and data downloads.

The gcCov database provides a dynamic visualization page to display the overall statistics of scientific data and to describe relationships between entities. Dynamic visualization is a great way to provide an overview of all CoVs or a specific virus category. Additionally, data visualization can easily be used to identify study objects by building a CoV knowledge graph. Details of visualization functions are described in Figure S1.

## Multiple search functions

In gcCov, a text‐based field search option allows users to explore the LOD using either single or combined metadata information. The input query retrieves all data containing the corresponding keywords in the metadata fields (e.g., virus categories, isolation sources, hosts, and submission or collection dates).

Data can be further visualized by selecting one or more records from the search results list. The data visualization displays all cataloged entities and relationships of the selected records in a dynamic picture to facilitate further knowledge discovery based on the underlying semantic network. Currently, in gcCov, nine categories of entities are integrated in the semantic web and available for visualization on the result page. These entities include virus category, taxon, virus strain, nucleotide sequence, protein sequence, structure, publication, patent, and antibody. The relationships between every pair of these entities are integrated and hence can be visualized on the result page. The result page also displays a statistical summary for each entity, keyword counts of publications, and numbers of publications each year. In the dynamic visualization box, any two points can be selected from the dynamic pictures and set as the start point and end point to automatically search for the relationship between the two points (Figure S2A).

## SPARQL data query and data download

LOD is used to promote linking and sharing of data results. Therefore, the resulting data can be explored using any semantic web browser and are available online through a SPARQL endpoint (Figure S2B). A wide range of example queries have been provided as demonstrations of how to search data using semantic queries. Finally, as an open database, all data are grouped by virus categories and can be freely downloaded in XML, JSON, CSV, TXT, and OWL formats for postprocessing and further analysis. Downloaded data may also be reused in concert with other LOD resources.

## Integrated data analysis pipeline

To promote interactive online virus and virome sequencing data analysis, we packaged viral analysis tools with Docker, assembled tools into pipelines, and integrated them into the National Microbiology Data Center BioCloud computing system (http://nmdc.cn/) as services presented in gcCov. The platform provides four analysis modules based on nucleotide/gene/protein sequences in gcCov database, including similarity search, genome annotation, phylogenetic analysis, and viral genome extraction from metagenomics sequencing data. Details of pipelines are described in Figure S3.

## Diverse data sources

Scientific data on CoV are widely distributed throughout various, usually publicly available resources. The most important resources include primary data, such as nucleotide and protein sequences, and protein 3D structure information. Primary data form the basis for investigations of virus mechanisms, variant evaluation, and vaccine and drug design. On the other hand, CoV literature and patents, which contain up‐to‐date observations and research results are also important references. To construct a semantic web of CoVs, the first step is extracting information from all the available original data resources (Table S1). In this database, based on the taxonomy of the CoV, nucleotide and protein sequences were automatically extracted from publicly available resources like GenBank^7^ and UniProt^8^. Protein 3D structure data were extracted from the PDB database^9^. CoV antibody information was downloaded from CoV‐AbDab^10^. Literature was extracted from PubMed using a set of query keywords. Patents were manually downloaded from public patent resources. Keywords extracted from literature and patents were reorganized into manually curated field categories, such as detection, diagnosis, prevention and control, antibody, drug therapy, and vaccine.

## Ontology and data processing

To accommodate the CoV data, we defined a set of ontologies containing 11 classes with 18 object properties and 42 data properties. For some entities with existing ontologies, we shared and reused property definitions. To ensure that data and relationships were comprehensively and precisely reflected in the schema, classes were described by data properties and relationships between classes were described by object properties. We separated all these CoV data into eight virus categories, seven (229E, OC43, SARS‐CoV, NL63, HKU1, MERS‐CoV, and SARS‐CoV‐2) belonging to HCoVs and the eighth representing all the other CoVs. CoV particles contain four main structural proteins: spike (S), membrane (M), envelope (E), and nucleocapsid (N) proteins^11^. Accordingly, we set protein categories as properties to group the proteins into these four categories. Countries and literature were also organized into groups for further analysis and statistical evaluation.

The key step of constructing a successful semantic web is to precisely establish crosslinks between entities. Details of the data crosslink relationship are provided in Figure S4.

After the schema was created, we mapped the name of the relational table to a Resource Description Framework (RDF) class node and the column names to RDF “predicates.” Correspondingly, the values for each record in the relational database were mapped to instances, and various original data resources were represented by “entities” and “relations” in the RDF schema.

Additionally, resources were provided with uniform resource identifiers (URIs) to promote crosslinking with other databases and for data reuse. URIs were used to set hyperlinks to the referenced resources on the web.

**Figure 1:**
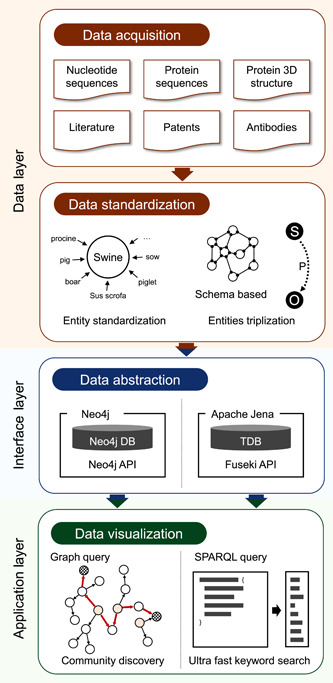
Pipeline of data processing and utilization. First, various nucleotide and protein sequences, three‐dimensional (3D) structure, literature, patents, and antibodies information were automatically extracted by a data collector or manually downloaded. The extracted data were integrated into an intermediate entities relationship database developed for gcCov after the data standardization process. Next, the data were transferred to a Resource Description Framework format using Jena and then used for SPARQL search. These data are also available for use with online search using Pubby for linked open data. The intermediate database was also integrated into a graph database (Neo4j) and then visualized using ECharts. The entire data‐processing workflow is automatically updated when any of its data sources are updated.

To properly accommodate information through the semantic web framework, we mapped different data resources to the RDF model and implemented a server for visualizing and publishing these data. Data processing schema is provided in Figure 1.

## CONCLUSION

The semantic web framework automatically establishes crosslinks between entities without human intervention. Thus, it is a powerful tool for displaying underlying relationships between research objects. gcCoV enables large‐scale data‐driven knowledge discovery and helps answer questions such as whether cross‐neutralizing antibodies exist among SARS‐CoV‐2, SARS‐CoV, and MERS‐CoV, considering that many of the SARS‐CoV‐1 and SARS‐CoV‐2 spike protein domains share high sequence and structural similarity. Therefore, the gcCoV system can provide clues to identify antibodies binding to multiple CoV antigens, which may be relevant to treat SARS‐CoV‐2 infections. This type of database is an important tool for the increasing information needs of CoV research, given its ability to mine text and data from previous studies and to provide clues for current prevention and treatment strategies. This is especially valuable after the outbreak of the COVID‐19 pandemic, where pathogenicity, immune response (including those associated with so‐called “long COVID”), drug discovery, and vaccine boosting require extensive and intensive evaluation.

## AUTHOR CONTRIBUTIONS

Wenyu Shi, Guomei Fan, Qinlan Sun, Juncai Ma, Yuanchun Zhou, Yuhai Bi, and Linhuan Wu planned and designed the project. Wenyu Shi, Haiying Yu, Siru Lin, Xiuqiang Sun, and Dongmei Liu created the data processing pipelines. Guomei Fan, Zhihong Shen, Chuan Hu, Zhen Meng, Siru Lin, Xiuqiang Sun, Xinjiao Zhang, and Qinlan Sun built the database and its web interface. Wenyu Shi, Qinlan Sun, and Linhuan Wu wrote the paper with inputs from all the authors. All authors read and approved the final manuscript.

## ETHICS STATEMENT

Not applicable.

## CONFLICT OF INTERESTS

The authors declare that they have no competing interests.

## Supporting information

Supporting information.

Supporting information.

Supporting information.

Supporting information.

Supporting information.
