# Supplementary figures and images for: gcCov: Linked open data for global coronavirus studies
Source: mLife. 2022 Mar 16;1(1):92–5. doi: 10.1002/mlf2.12008 (PMC9088579; doi:10.1002/mlf2.12008)

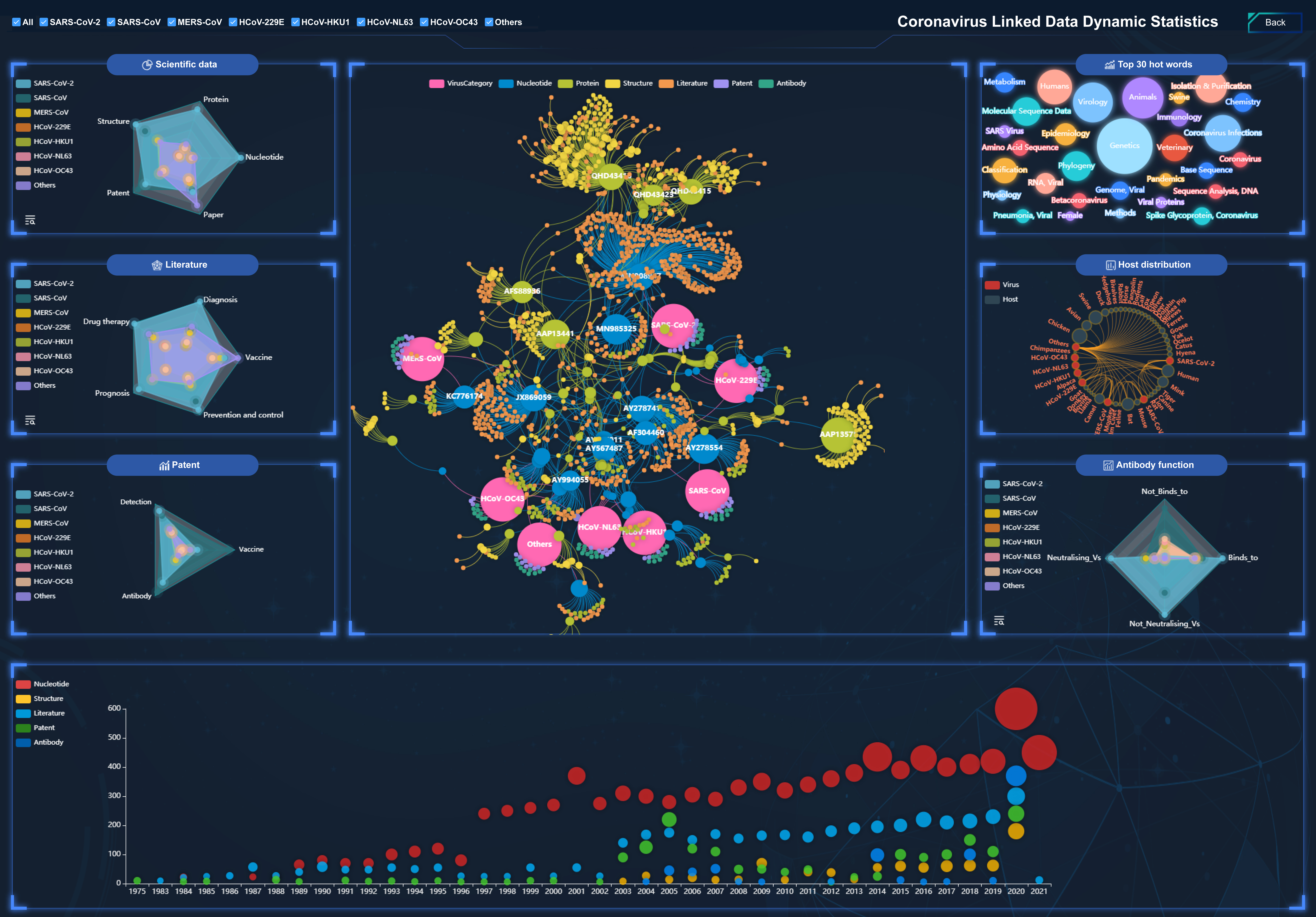

Supplement: Supplementary file 1 — Supporting information. [file MLF2-1-92-s004.jpg]

## Slide 1
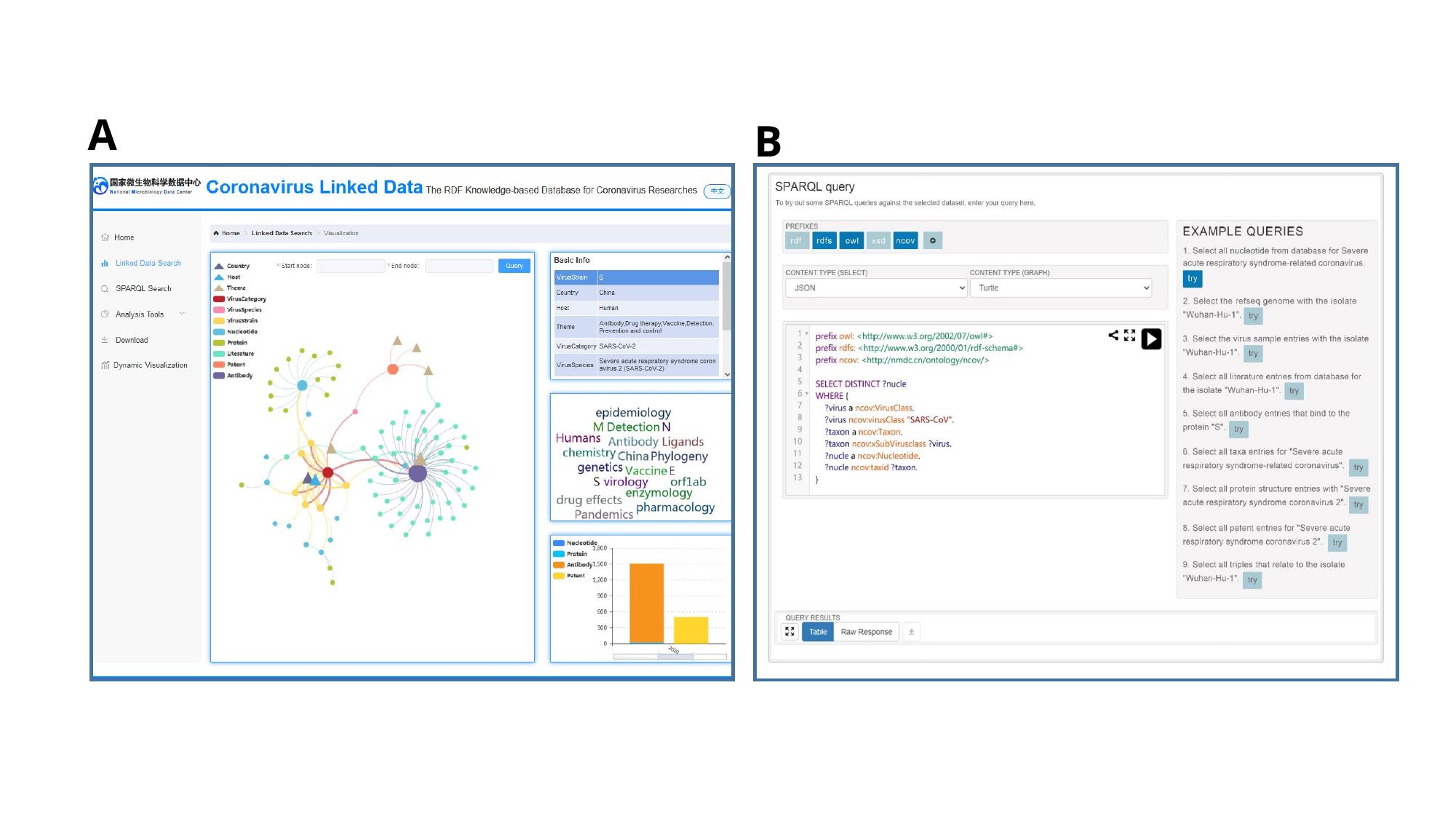

A
B

Supplement: Supplementary file 2 — Supporting information. [file MLF2-1-92-s003.pptx]

**A**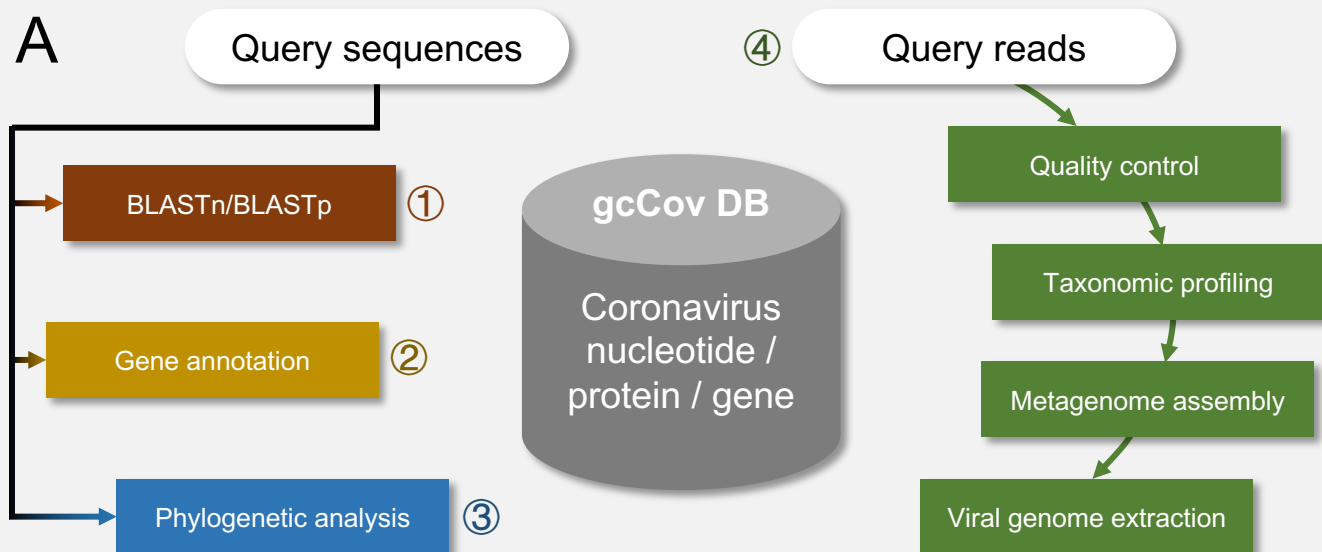**B**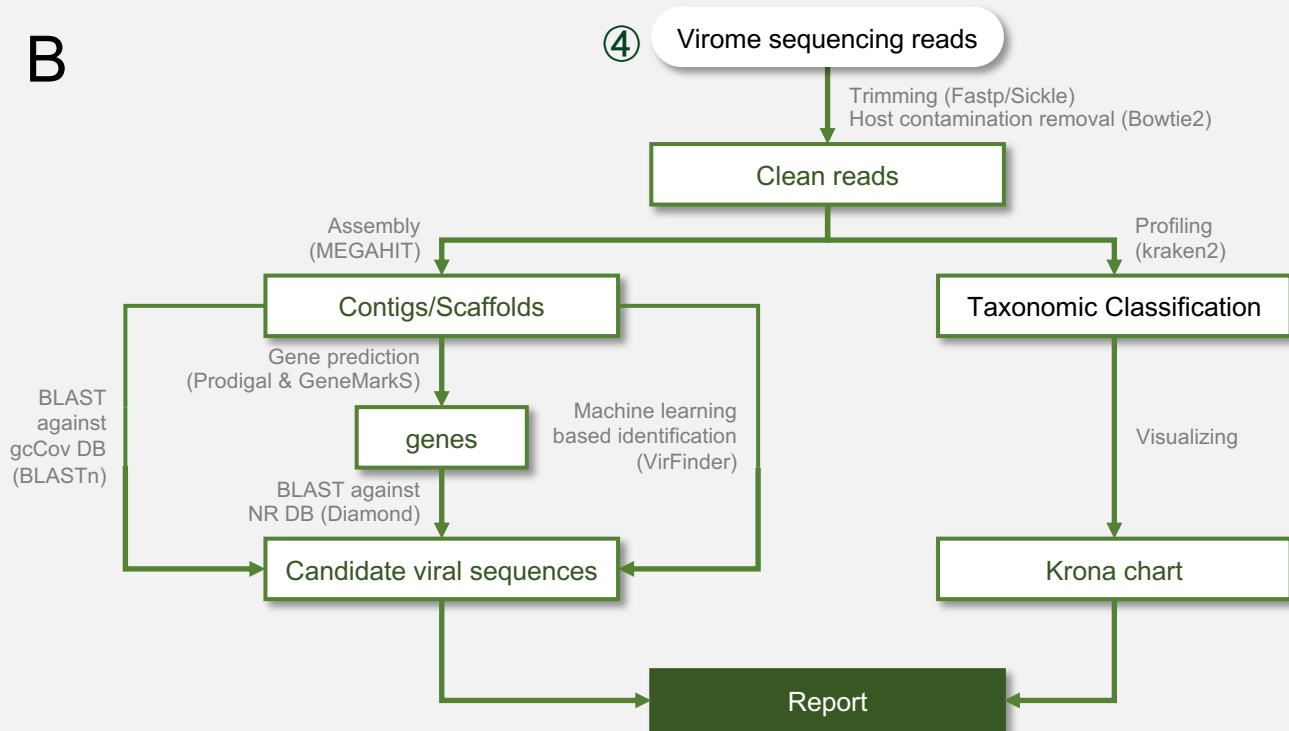

Supplement: Supplementary file 3 — Supporting information. [file MLF2-1-92-s005.pdf]
