# Supplementary material for: gcCov: Linked open data for global coronavirus studies
Source: mLife. 2022 Mar 16;1(1):92–5. doi: 10.1002/mlf2.12008 (PMC9088579; doi:10.1002/mlf2.12008)
Supplement: Supplementary file 4 — Supporting information. [file MLF2-1-92-s001.pptx]

## Slide 1
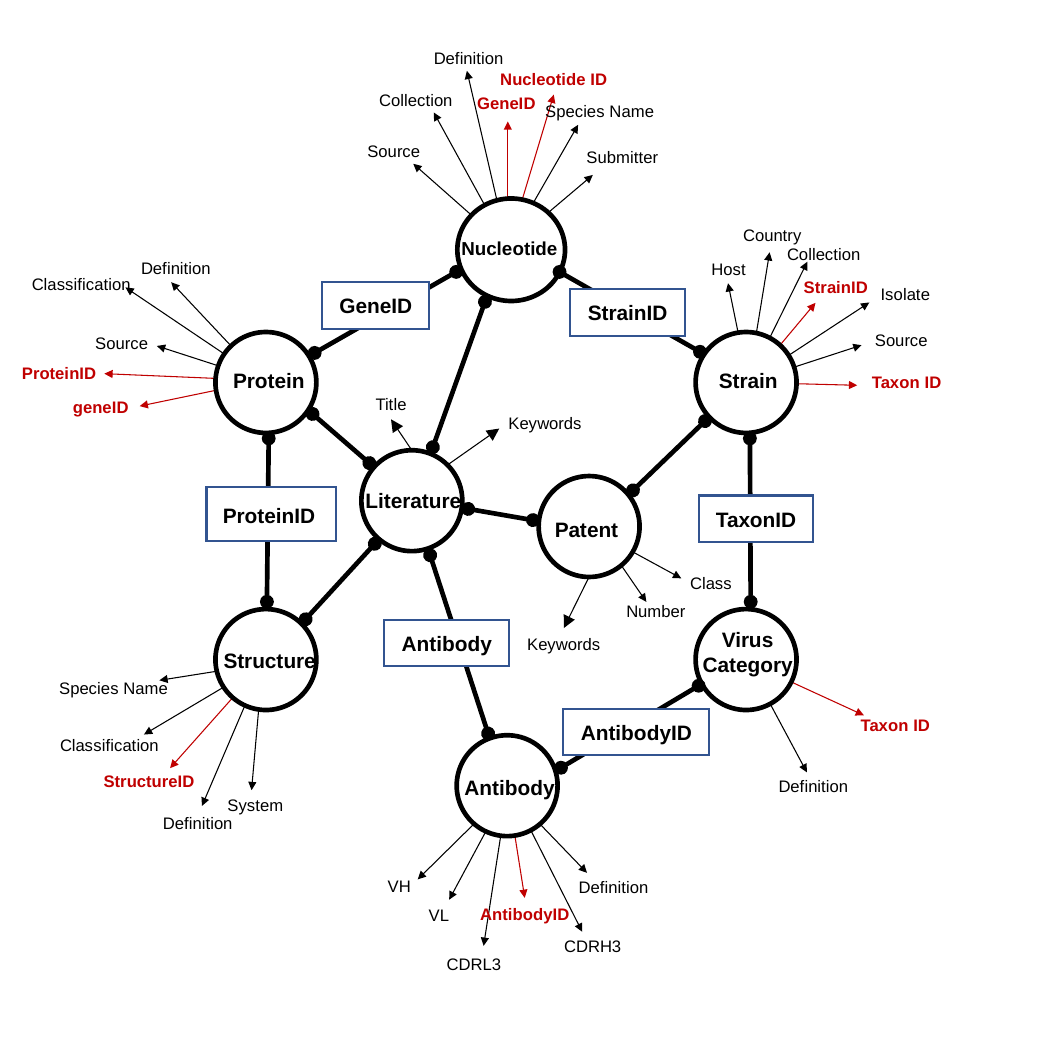

Definition
Nucleotide ID
Collection
GeneID
Species Name
Source
Submitter
Country
Collection
Host
StrainID
Isolate
Source
Taxon ID
 Nucleotide
Definition
Classification
Source
ProteinID
geneID
GeneID
StrainID
Protein
Strain
Title
Keywords
Literature
ProteinID
TaxonID
Patent
Class
Number
Virus
Category
Antibody
Keywords
Structure
Taxon ID
Definition
Species Name
Classification
StructureID
System
Definition
AntibodyID
Antibody
VH
Definition
AntibodyID
VL
CDRH3
CDRL3
