# Supplementary material for: gcCov: Linked open data for global coronavirus studies
Source: mLife. 2022 Mar 16;1(1):92–5. doi: 10.1002/mlf2.12008 (PMC9088579; doi:10.1002/mlf2.12008)
Supplement: Supplementary file 5 — Supporting information. [file MLF2-1-92-s002.docx]

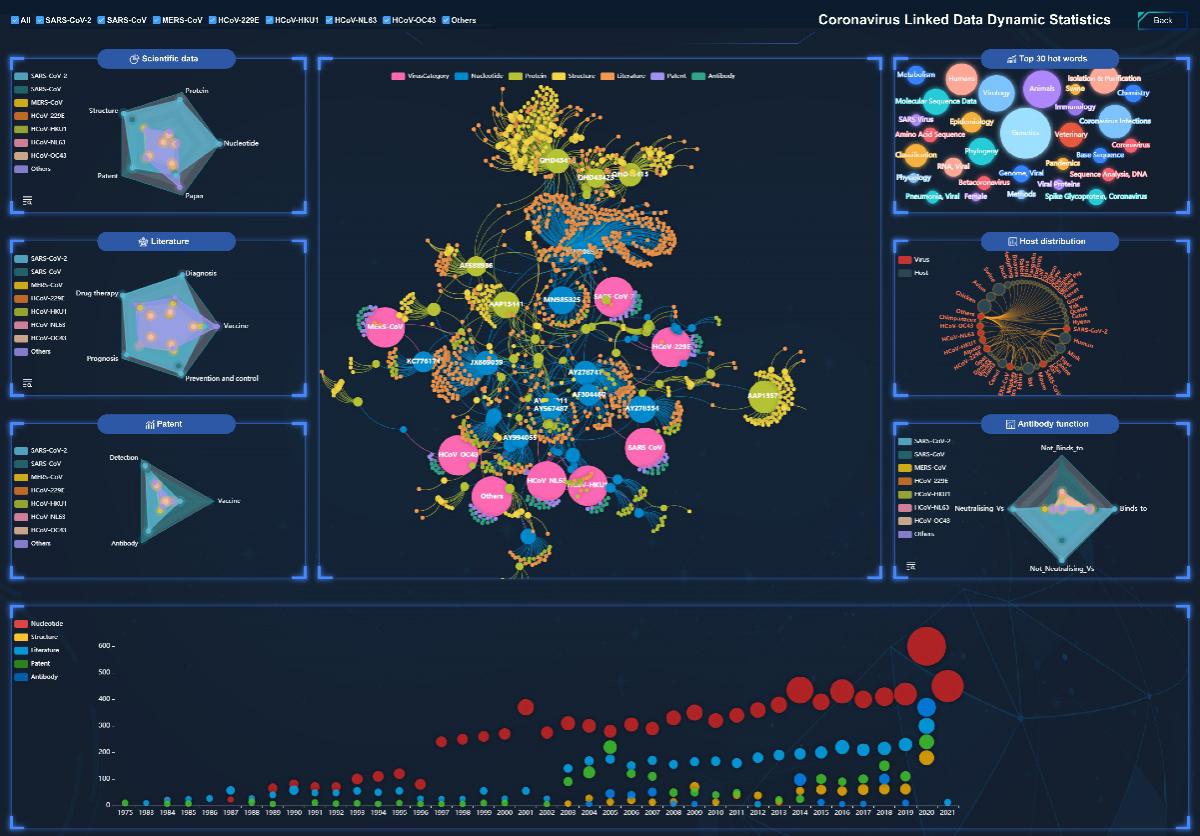


Figure S1. Visualization of the linked open data. The graph in the middle is an interactive interface displaying an overview of the relationship among prominent entities in the database. Users can easily identify the most important strains, publications, genomes and their connections with other research results. The section to the left provides statistics of all scientific data, literature types and patent types. The distribution areas indicate published research and the results in different virus categories organized by different subjects. The top section at right provides keyword counts reflecting the current focus, issues, and trends of the selected virus category. The middle section at right presents host distributions, and the bottom section displays antibody function analysis, which are also based on the selected virus category. Although the exact mechanisms of genetic recombination are still unclear, CoVs circulating between multiple host species may lead to increase of recombination events. Host distribution graph indicates that CoVs have an extensive range of hosts. Some CoVs share the same hosts, which may indicate infection of a common ancestor or inter-species transmission and recombination events. Global research has revealed more than 1800 antibodies for CoV infection. The antibody function graph displays an antibody's response to different CoV infections. These types of graphs are helpful for determining whether an antibody provides effective resistance against a particular CoV strain. The bottom part displays annual data of nucleotide sequences, publications, patents and antibodies.


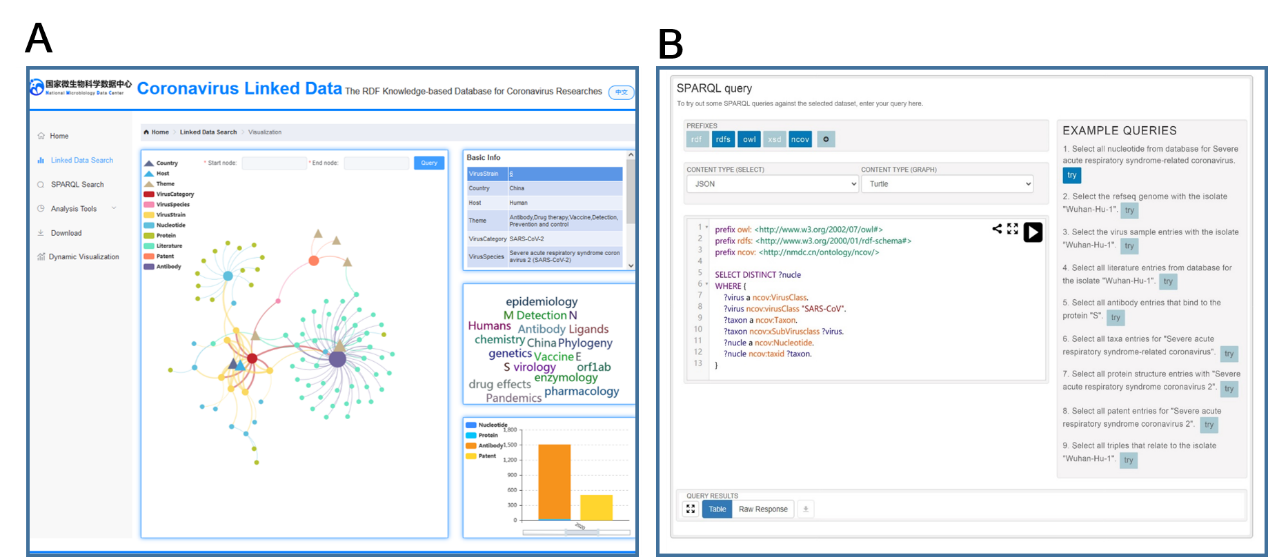


Figure S2. User interface and search functions. (A) Dynamic visualization page. (B) SPARQL data query demo. In the linked data search part, users could input their query by using one or several meta data values as filter, after that, a list of results will display. Users could further select interested results and add them into visualization. Finally, users will get a result with dynamic visualization of all selected sequences. For example, we searched all SARS-CoV-2 whole genome sequences isolated from Wuhan, China, and then selected several genomes to be displayed. The visualization page displayed all these selected genomes and their related protein sequences, protein structures, literature and antibody information in an interactive page. If any two points were selected as start point and end point, the system will automatically find all possible links between these two points. In the SPARQL data query page, we provide demo for searching the database by SPARQL such as selecting all nucleotide sequences or literature with some specific characteristics.


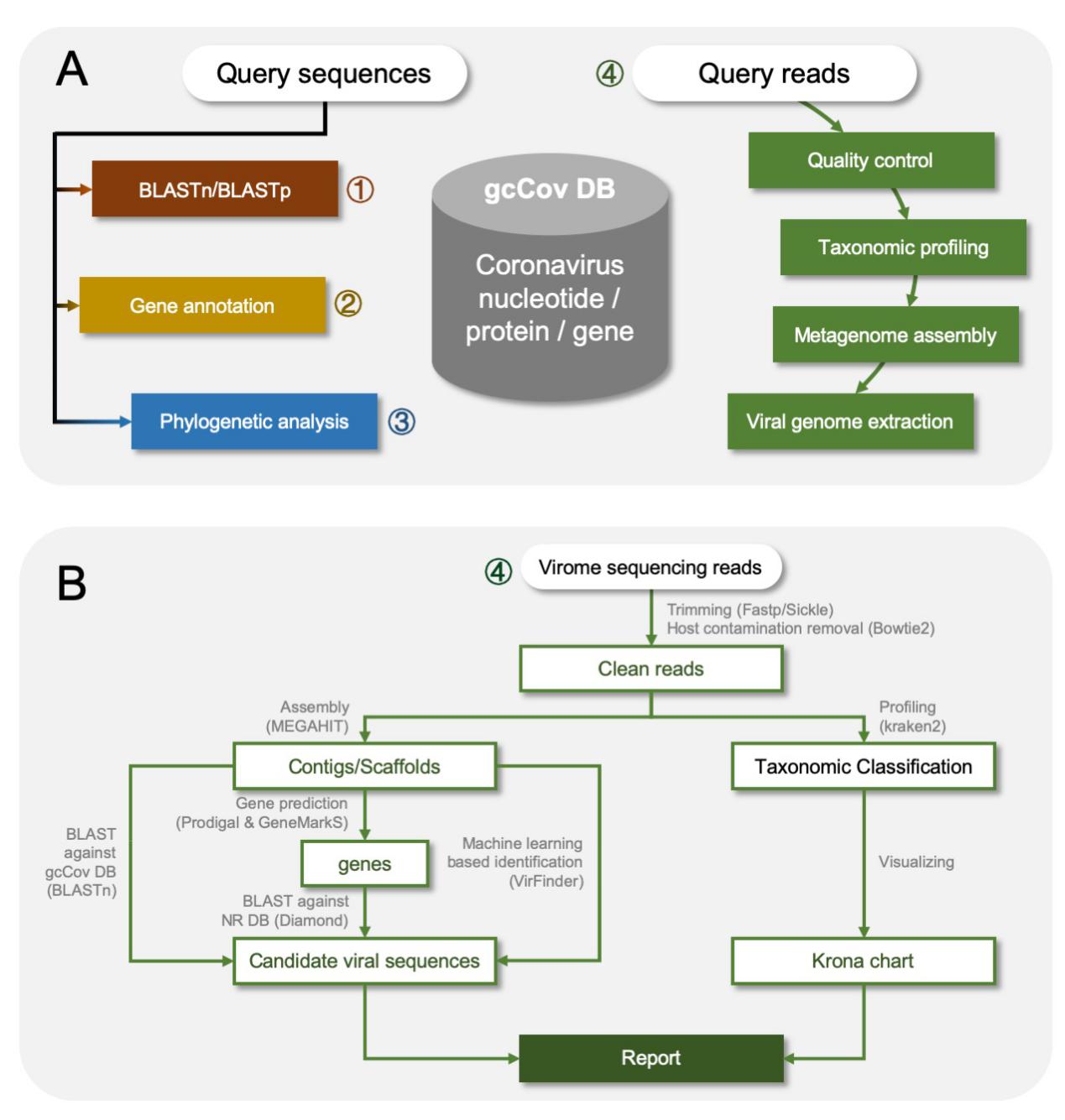
 In panel B, “genes” in the box should be changed to “Genes”.

Figure S3. Data analysis pipelines integrated in gcCov. (A) Four pipelines for sequence similarity search, genome annotation, phylogenetic analysis and viral genome extraction from metagenomics sequencing data. (B)Data processing workflow of viral genome extraction pipeline. Firstly, the local alignment module enables users to submit coronavirus sequences to search against the gcCov sequence database using BLASTn or BLASTp. After that, the viral genome annotation module enables users to submit coronavirus genome to perform gene prediction and automated annotation using GeneMarkS(1), Prokka(2) or VAPiD(3). The viral sequence phylogenetic analysis module enables users to submit coronavirus sequences and select a set of sequences to perform phylogenetic analysis. The submitted sequences are aligned with one another using MAFFT(4), and maximum likelihood phylogenies are constructed using FastTree(5). Finally, the viral sequence extraction module is designed for viral genome recognition in metagenomic or meta-transcriptomic sequencing project aiming at coronavirus detection. Firstly, raw reads are trimmed into clean reads and host contamination in the data are removed by mapping all reads to host reference. Then, taxonomic profiling is estimated and visualized by Krona(6) interactive chart. At the same time, all the clean reads are assembled into contig/scaffold. Next, viral sequences are identified as candidate through three ways: coronavirus sequences are identified by BLAST against gcCov genome database; other virus sequences are identified by the gene similarity against NR database; another machine learning identification is performed by VirFinder(7) to detect novel virus sequences. Finally, the coverage of the candidate viral sequences is calculated and all sequences are annotated. Results are reported to the users after all analysis procedures are completed.


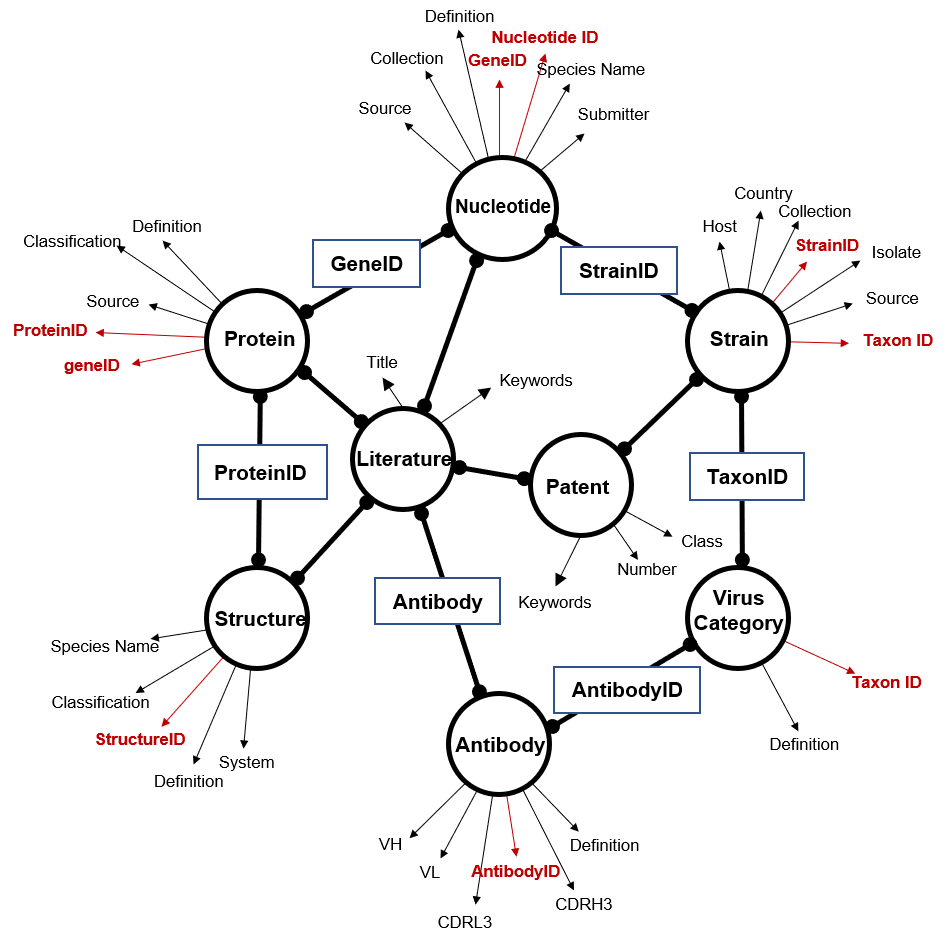


Figure S4. Schematic of classes and relationship of gcCov. In our case, virus categories were linked with virus strains using taxonomy information. NCBI taxonomy ID was used to establish links. Virus strains were used to identify individual isolates or samples from environment extracts. Each virus strain may have one or more nucleotide sequences. Nucleotide sequences are linked to protein sequences by the genes they encode. The literature was linked with nucleotide sequences, protein sequences, and protein 3D structure by automatically scanning the accession numbers of these three types of data. Antibody and patent information were included in this semantic web based on their associated virus categories. The overall schema of the database was created by mapping their relationships with external links.

Table S1: Data sources integrated in gcCov

| **Data type** | **Data resource** | **Triplet count** |
| --- | --- | --- |
| Nucleotide sequences | NCBI GenBank, Gisaid | 1,186,843 |
| Protein sequences | NCBI protein, UniProt | 2,177,531 |
| Protein 3D structure | PDB | 1503 |
| Antibody | CoV-AbDab | 1837 |
| Literature | PubMed, PMC | 4465 |
| Patents | World Intellectual Property Organization (WIPO) | 2077 |
| Keywords | Author generated | 58,793,871 |
| Total |  | 62,168,127 |

All data were collected up to May 2021.

References

1. Besemer, J, Lomsadze, A, Borodovsky, M. GeneMarkS: a self-training method for prediction of gene starts in microbial genomes. Implications for finding sequence motifs in regulatory regions. *Nucleic acids research*. 2001; **29**(12): 2607-18.

2. Seemann, T. Prokka: rapid prokaryotic genome annotation. *Bioinformatics*. 2014; **30**(14): 2068-9.

3. Shean, RC, Makhsous, N, Stoddard, GD*, et al.* VAPiD: a lightweight cross-platform viral annotation pipeline and identification tool to facilitate virus genome submissions to NCBI GenBank. *BMC bioinformatics*. 2019; **20**(1): 1-8.

4. Katoh, K, Kuma, K-i, Toh, H*, et al.* MAFFT version 5: improvement in accuracy of multiple sequence alignment. *Nucleic acids research*. 2005; **33**(2): 511-8.

5. Price, MN, Dehal, PS, Arkin, AP. FastTree 2–approximately maximum-likelihood trees for large alignments. *PloS one*. 2010; **5**(3): e9490.

6. Ondov, BD, Bergman, NH, Phillippy, AM. Interactive metagenomic visualization in a Web browser. *BMC bioinformatics*. 2011; **12**(1): 1-10.

7. Ren, J, Ahlgren, NA, Lu, YY*, et al.* VirFinder: a novel k-mer based tool for identifying viral sequences from assembled metagenomic data. *Microbiome*. 2017; **5**(1): 1-20.
